# Round the Hospitals

**Published:** 1919-08-02

**Authors:** 


### ROUND THE HOSPITALS.

The Buckingham Palace Gardens have never witnessed a more picturesque and interesting gathering than that which the King and Queen assembled on July 25 from among the ranks of war workers. The guests numbered 10,000, and it was a triumph of organisation that there was no crowding or confusion either during the party or in getting these vast numbers safely in and out of the gardens. The King was in field marshal's uniform, and the Queen wore a dress and toque of hyacinth blue, embroidered in silver. The Prince of Wales, Prince Albert, and Prince Henry wore the uniform of their respective branches of the service, and Princess Mary was in V.A.D. uniform, out of compliment to other war workers and the nursing sisters. The Royal circle included also the Duke of Connaught, Lord and Lady Athlone, Princess Christian, Princess Helena Victoria, Lady Patricia Ramsay, and the Crown Prince and Crown Princess of Sweden. The King and Queen received the workers, both individually and in companies, in their pavilion. The nursing services represented numbered all the naval, military, and Air Force organisations, together with many civilian hospitals and associations. The R.A.M.C., the B.R.C.S., and the St. John Ambulance Association all had many members present.

Many nursing sisters had the honour of presentation to the King and Queen, and among these was Miss Beadsmore Smith, the new matron-in-chief of Q.A.I.M.N.S., and her colleague, Miss Dowse, who was on the *Britannic* when it was torpedoed, and subsequently did fine service in Taranto. Dame Sidney Browne, R.R.C., was conspicuous among a large group of Territorial sisters; Miss Cox-Davis, R.R.C., and Miss Lloyd Still, R.R.C., were both present. There was a large contingent of B.R.C.S. sisters, as well as V.A.D.s, and the uniform of the F.A.N.Y.s was easy to distinguish. Among the women conspicuous for war work might be seen Dame Katharine Furse and Dame May Whitty, Lady Ampthill, Lady Oliver, Mrs. St. Clair Stobart, and Lady Cowdray. But individual recognitions were very difficult, on account of the sheer wealth of good women scattered on the lawns. It

**ROUND THE HOSPITALS**—*(continued)*.

might almost be said that everybody who was anybody could have been encountered among the animated groups which aspired chiefly for a sight of their host and hostess and found all their other wants anticipated in the large marquees.

At the Investiture held at Buckingham Palace on July 26, the King personally conferred decorations on members of the nursing services as follows:—Royal Red Cross, First Class: Sister Alice Walpole, Q.A.I.M.N.S.R.; Sister Jessica Freshfield, B.R.C.S.; and Matron Rachel Fogarty, South African M.N.S. Royal Red Cross, Second Class: Sister Mabel Kirkpatrick, Q.A.I.M.N.S.R.; Matron José Walker, B.R.C.S.; Matron Alice Messum and Miss Elizabeth Urquhart, Civil N.S.; Miss Dorothy Greig, Miss Helen Harrison, Miss Isabel Montford, Miss Nessie Warner, Miss Gertrude Whitehurst and Miss Violet Wotton, V.A.D.; Miss Margaret Davidson and Miss Katharine Fabling had the honour of receiving the Military Medal from His Majesty. All these ladies were afterwards received by Queen Alexandra at Marlborough House.

Girton College, Cambridge, celebrated its Jubilee on July 26, when a garden party was given, at which the President of the Board of Education and many notable guests were present. Among distinguished Girtonians are many who have made their mark in practical directions. Miss Adelaide Anderson, C.B.E., is H.M. Principal Lady Inspector of Factories, and Miss F. H. Durham, O.B.E., is Chief Woman Inspector to the Ministry of Labour, facts which speak for the value of collegiate training as preparation for public service. The College is proud also of Dr. Lilian Chesney, who was among the first to volunteer for medical duty in Serbia. One former student, Miss M. E. Vernon-Harcourt, became matron of a hospital at Etaples, and another, Miss G. L. Buckley, devoted herself to work in France as an *x*-ray operator. A band of Special Service Red Cross Workers did good work in one of the military hospitals at Cambridge under the superintendence of Miss Clover, the College Secretary. We may be permitted to hope that in the course of the next fifty years the College may extend its activities to cover a nursing diploma.

Exeter Cathedral was the scene of a very impressive ceremonial on July 11, when over a thousand nursing sisters, Red Cross nurses, and ambulance workers gathered from all parts of Devon to take part in commemorating those who had given their lives in the war, and in giving thanks for the declaration of peace. The county of Devon was the first to get its hospitals into working order after the declaration of war, and the first patients were received in October 1914. Thirty-four hospitals were organised and 3,905 beds equipped. The number of patients received was over 45,000, and the *personnel* engaged totalled 2,735. The organising abilities and great personal gifts of the county director, Mr. J. C. S. Davis, C.B.E., were warmly brought to notice by the Lord Lieutenant of the county, Earl Fortescue, and his popularity was displayed in the gifts of a silver salver and a motor-car, subscribed for by his fellow-workers in every part of the county. The note sounded in all the speeches, starting with the Dean's sermon in the cathedral, was the future of valuable service which lay before the voluntary organisations now that war is ended.

Mrs. Lloyd George opened some fine new day nurseries at Bethnal Green on July 24, and a large company gathered at the Town Hall under the Lord Mayor to celebrate the occasion. Two old houses overlooking Victoria Park have been remodelled for the use of babies from one month to five years. There are fifty clients already at this babies' hotel de luxe, who pay 7d. a day and get three meals. Lady Islington had some very sensible things to say, explaining that it was no more "charity" in the wrong sense of the word to found such institutions than to found Eton College. She urged the need for working mothers, as well as for rich mothers, to get the children off their hands for a time while they got through their daily round, and ably defended her patrons from the charge of putting their children in a crêche in order that they might enjoy themselves.

Guy's is one of the few hospitals which can chronicle in its annual report the fact that "a steady influx of new probationers has maintained the nursing service at full strength." The new scale of salaries is £12, £16, £22. A large extension of the Nurses' Home is in contemplation, giving forty-one extra bedrooms to meet the increase in nurses rendered necessary by alterations in the time-table and by the addition of new wards. The cost of the new block, which is to have ample space on the lower floors for the new departments of massage, Swedish remedial exercises, and medical electricity, was reckoned, at pre-war prices, to amount to £30,000. The massage school has a very high reputation, and its rapid growth during the war has made enlarged quarters an imperative necessity. Fifty-six pupils obtained the massage certificate last year, nine the certificate for Swedish remedial exercises, and one the teacher's certificate. On three occasions Guy's students headed the pass list for all England.

A "Hospital Sister," writing to us on the question of a protected out-door uniform or badge for nurses, argues against the need for the profession to be labelled "any more than that of other women." Admitted that outdoor uniform is convenient and saves expense, it is contended that under the improved conditions of work and pay in the nursing services, hospital nurses, at any rate, might discard cloak and bonnet and appear in mufti. Our correspondent is distressed at the

**ROUND THE HOSPITALS**—(*continued*).

criticisms levelled against trained nurses "because of the behaviour of young women who choose to wear what they evidently think a fascinating uniform." But the only real remedy for this abuse is to press for a protected uniform to be worn by registered nurses only. Once that were conceded all persons wearing nurse's uniform of the wrong pattern would be self-exposed as either imperfectly trained or impostors.

THE suggestion put forward in these columns that there is need for a special sanatorium for nurses attacked by phthisis, has brought us an interesting communication from a nurse who has had bitter experience in her own person of nurses' sufferings in respect of this disease. As an insurance patient this lady procured admission to a sanatorium after waiting two months, but could only be retained for a period of four months. Her home is in a manufacturing town, and she has no alternative but to return to it. The disease is only in the early stage, and under suitable conditions and a prolonged course of treatment complete recovery is probable. We again urge most strongly on the organisations concerned with nurses' welfare the imperative need for founding and partly endowing a house of recovery for tuberculous nurses. Although the conditions under which nurses work are eminently healthy, there are various causes which contribute to break down their resistance to the disease. Early and long-continued treatment would save many women whose lives have been devoted to others.

A WARRANT OFFICER, R.A.S.C., writes to the *Daily Telegraph* to remind the public of the services rendered by the Army Sisters, "the most noble women who ever graced God's earth." "The Army nurses, in many instances, have given their all. They left their home comforts for unknown dangers. They endured gunfire and bombardment. They witnessed most appalling sights of mutilated men, and have nursed and soothed them through their death agonies. Many in the present day are broken in health, owing to the strain of hardships, and many have proved themselves faithful unto death, as the cemeteries in France and Flanders testify." The "Warrant Officer" suggests that at least one bed in every women's ward in every hospital of the country should be endowed and dedicated to their memory, that they be named "Our Sisters' Beds," and that a memorial service be held annually in every hospital with beds so endowed.

DURING the influenza epidemic last year nine of the Queen's Nurses succumbed to the disease. The services they rendered and the self-sacrifice displayed by them and by village nurses throughout the country are acknowledged with pride by the Council of Q.V.J.I.N. But it is not enough to put on record their appreciation of the nurses' devotion. There should be an organised effort throughout the country to render it impossible for the future that one woman, unaided, should be left to stem the torrent of an epidemic. The history of infectious disease makes it clear that the country is exposed to the danger of a recurring wave of influenza, and that it may be here within a few months. Places which were lightly visited last year may receive the full force of the next blow. And no place is secure against the infection.

So far as nursing is concerned, all that was possible during the influenza outbreak last autumn was to keep people in bed and supply their need for hot, nourishing drinks. When whole families were stricken this needed an organised group of voluntary workers, visiting, under the directions of doctor and nurse, two or three times a day the cases reported to headquarters, in order to carry round beef-tea and hot milk from a central kitchen. Where this was done the nurse was set free to give her aid in really serious cases, and her energies, though severely taxed, were not uselessly broken down by attempting the impossible. We would seriously urge upon the county nursing associations and on Queen Victoria's Jubilee Institute the need for rousing nursing associations throughout the country to prepare supplementary aid of this description for their nurses in view of the probable recurrence of the epidemic.

A VERY painful impression has been created in Liverpool by the tragic death of Alice Kate Jones, aged twenty-six, a recently certificated member of the nursing staff at the David Lewis Northern Hospital. Late on the evening of July 24 the porter heard shots in the vicinity of the hospital, and on investigation discovered the body of Nurse Jones lying on the footpath close by. Life was quite extinct. A Canadian soldier named Joseph Roy Hutty surrendered himself to the police, and was brought before the magistrates next morning. On being charged he said: "I shot Nurse Jones; I only hope she is dead." It appears that he was treated at the hospital last year for shell-shock, and sent back to Canada. A few weeks ago he reappeared and asked for Miss Jones.

IT would seem from the report recently issued of Lady Minto's Indian Nursing Association that nursing sisters desirous of taking service in India would be welcomed by that organisation. All through 1918, and particularly during the first half of the year, the branches were very short-handed, partly owing to the fact that the number of patients nursed exceeded by 196 all previous records. The influenza epidemic brought a period of incessant stress, and the devotion of the sisters was warmly appreciated by the Europeans. New departments of activity are in course of development by the Society, notably the establishment of a second nursing home for maternity cases in Simla. Candidates should apply for a form to fill in to Dame Sidney Browne, 80 Pall Mall, S.W. 1. Preference is given to fully trained applicants who hold certificates in special subjects,

**ROUND THE HOSPITALS**—(*continued*).

such as nursing of tropical diseases, fever, massage, etc. All should hold midwifery certificates.

It is stated that Miss Annie Bacon, health visitor to the Southwark Borough Council, is in danger of losing her appointment, owing to her refusal to wear the uniform of a trained nurse, which the Council insist upon as a condition of service. Miss Bacon contends that she is not a trained nurse, and therefore is not entitled to assume this garb. She would be willing to wear a uniform agreed upon for health visitors. It is greatly to be desired that the next step taken towards consolidating the profession of health visiting should be choice of a suitable outdoor uniform which is not that of the trained nurse.

The Secretary of State for India requests us to state the number of applications already received for temporary appointment to Q.A.I.M.N.S. exceeds the vacancies, and that no more applications can be accepted at present. Further vacancies will be announced as they arise.

The American Red Cross Society appointed May 7 as the National Mémorial Day for Miss Jane A. Delano, who died last April at Base Hospital No. 69 in Savenay as the result of her faithful labours for the wounded, and we have lately received accounts of the celebration. Miss Delano was formerly superintendent of nurses at Bellevue, New York, then chief nurse of the Army Nurse Corps, and finally director and organiser of the department of nursing of the American Red Cross. Her services to her country in this connection can hardly be exaggerated and, as so often happens in the nursing world, her genius for practical detail and exact organisation was combined with a wide outlook and a very illuminating personality. Her international work in Europe won her a host of friends and none came within the sphere of her influence without feeling the force of her inspiration. Her department was one of the conspicuous successes of the United States in the War.

The Society for the Propagation of the Gospel in Foreign Parts has not escaped the trials of the other missionary societies resulting from deficiencies in the staff. Their medical missions, in particular, have suffered. The reports received from the different stations are a record of the valiant efforts of greatly depleted staffs to keep things going with a constant straining after more volunteers. At the moment the work has probably reached its lowest ebb since medical missions were founded, and the time is ripe for a complete reconstruction under modern theories. To enable hospitals which were closed down to be reopened, seven medical men and seven nursing sisters are urgently needed, while over forty medical missionaries must come forward if the schemes of the various Bishops are to be carried through. We foresee a danger that the Society may be moved to open more hospitals than can be properly staffed. It is merely throwing valuable lives into the melting-pot to open with "a skeleton staff," provide for no reliefs, and trust to luck.

Newcastle has inaugurated a "Cavell Memorial Fund" for the Northern Counties of England, at a meeting held under the presidency of the Lord Mayor. The object is to provide annuities and gratuities to nurses in necessitous circumstances. Sir Thomas Oliver exposed the need for assisting trained hospital nurses who through illness or age were unable to follow their vocation. The Committee offered towards a fund for this purpose £850 Victory War Bonds as a lead-off, and numerous other subscriptions were promptly handed in, including one of 2s. 6d. from a demobilised soldier, just returned from abroad.

The Birmingham District Nursing Society is in great straits for lack of money, but the city cannot do without it, and will have seriously to set about raising a regular income to meet the deficit of £1,000 a year in its finances. During the influenza epidemic, in which there were 450 cases and seventy-six deaths, the Society fairly saved the situation for the Health Committee. Mr. Cadbury, who praised its efficiency at the annual meeting, told the Lord Mayor and the subscribers that there was going to be a higher standard of nursing and an inquiry into remuneration and hours. We have not the slightest doubt that when the nurses' remuneration is brought up to a proper level and their working hours decreased the annual sum needed to keep the Society solvent will be found to exceed by a high figure the present thousand-pound deficit.

A project is on foot at the Stoke Poor Law Hospital for fitting up a portion of the building for the use of private patients able to contribute to the cost of their treatment. It is a very interesting departure, and will, if found practicable, bring a fresh current of interest and variety into the nursing department which will be good for everyone concerned.

A very unorthodox remark, "I think that nurses are equal to doctors," was made by Lord Roe in presiding at the annual meeting of the Royal Derby and Derbyshire Nursing and Sanitary Association. He made matters right by explaining that "good nursing very often takes the place of medicine," a proposition few would dispute. The names of many of the staff, including eleven sisters who have distinguished themselves in war-work, for the most part abroad, are recorded in the report, and those who stayed at home were not forgotten. Nurse Kilbourne was congratulated on the completion of twenty years' service with the Association. Excellent results were obtained in the training centre, from which thirty-two midwives passed the C.M.B. examination. It is very satisfactory to learn that £6,000 has been set aside to form the nucleus of a pension fund.

